# A physical therapist and nurse practitioner model of care for chronic back pain using telehealth: Diagnostic and management concordance

**DOI:** 10.1177/1357633X221098904

**Published:** 2022-05-12

**Authors:** Stacey Lovo, Liz Harrison, Megan E O’Connell, Thomas Rotter, Brenna Bath

**Affiliations:** 1 School of Rehabilitation Science, 7235 University of Saskatchewan, Saskatoon, Canada; 2 College of Arts and Science, 7235 University of Saskatchewan, Saskatoon, Canada; 3 School of Nursing Health Quality Program, 4257 Queen’s University, Kingston, Canada

**Keywords:** Telerehabilitation, virtual care, low back pain, physiotherapy, telehealth

## Abstract

**Introduction:**

Virtual care using videoconference links between urban-based physical therapists and nurse practitioners in rural primary care may overcome access challenges and enhance care for rural and remote residents with chronic low back disorders (CBD). The purpose of this study was to evaluate the concordance of this new model of care with two traditional models.

**Methods:**

In this cross-sectional study design, each of 27 participants with CBD were assessed by: 1) a team of a nurse practitioner (NP) located with a patient, joined by a physical therapist (PT) using videoconferencing (NP/PT_team_); 2) in-person PT (PT_alone_); and 3) in-person NP (NP_alone_). Diagnostic and management concordance between the three groups were assessed with percent agreement and kappa.

**Results:**

Overall diagnostic categorization was compared for PT_alone_ versus NP_alone_ and NP/PT_team_: percent agreement was 77.8% (*k* = 0.474, *p* = 0.001) and 74.1% (*k* = 0.359, *p* = 0.004), respectively. In terms of management recommendations, the PT_alone_ and NP_alone_ demonstrated strong agreement on “need for urgent surgical referral” (92.6%, *k* = 0.649 (*p* < 0.00) and slight agreement for “refer to primary physician for pharmacology, lab or imaging” (81.5%, *k* = 0.372 (*p* = 0.013). The PT_alone_ and NP/PT_team_ demonstrated strong agreement on “need for urgent surgical referral” (96.3%, *k* = 0.649, *p* = 0.000) and “recommendation for PT follow up” (88.9%, *k* = 0.664, *p* = 0.000).

**Discussion:**

The diagnostic categorization and management recommendations of the team using videoconferencing for CBD were similar to decisions made by an in-person PT. This model of care may provide a method for enhancing access to PT for CBD assessment and initial management in underserved areas.

## Introduction

Chronic low back disorders (CBD) are a prevalent and costly health problem that disproportionately impacts rural and remote residents. Twenty percent of Canadians have CBD and those living in rural and remote areas are 30% more likely to have CBD.^1^ In the year 2014, Bone and Joint Canada estimated that 6–12 billion dollars per year were spent on CBD in Canada, not including time lost at work.^2^ Not only costly to individuals, CBD strain health care resources because of high rates of primary physician care visits,^3,4^ specialist consultations, and diagnostic procedures.^5,6^

CBD are often multifactorial, including functional disabilities and psychological issues.^7^ Physical Therapists (PTs), whose specialized knowledge of musculoskeletal conditions may exceed that of most physicians (with the exception of orthopedic surgeons),^8^ have much to offer for improving appropriateness and effectiveness of CBD care. One example is that PTs have been shown to improve musculoskeletal management via triage, and spinal triage can in turn improve orthopedic surgery wait lists.^9,10^ PTs can play an important role in primary care for CBD and may facilitate reduction of wait times for diagnostics and specialist care.^10^ This role could have great potential for health care system savings. However, in rural regions such as the Canadian province of Saskatchewan, there is reduced access to PT, especially to those with focused musculoskeletal knowledge.^11,12^ Although there has been much discussion in Canada about the need for interprofessional teams in primary health care service delivery models,^11^ the involvement of PTs in such teams is rare.^12^ Lack of access to appropriate CBD care in primary health care is exacerbated in many rural and remote communities.^13–15^

The use of virtual health technologies, such as telehealth or secure videoconferencing, is a promising means to help improve access to PT services in rural settings.^16^ Although videoconferencing is effective for conducting a patient interview,^17^ performing an effective physical examination via this medium is a barrier perceived b in the adoption of virtual services.^18^ The primary concern is that elements of a conventional face-to-face physical examination requires the PT to perform hands-on procedures with the patient; ^18^ a ‘hands-on’ assessment cannot be achieved via videoconferencing, so adaptation of the conventional uniprofessional (i.e. PT only) assessment must occur. Previous research has examined the validity of individual components of a PT back pain assessment over videoconferencing versus in person assessment. Many components of a neuromuscular assessment require someone to be with a patient at the videoconferencing end to conduct specific tests. Therefore, a novel approach is required to overcome traditional barriers associated with a ‘hands-on’ PT assessment. In rural Saskatchewan, NPs are community primary health care providers in rural and remote regions where physician services are limited and this has been shown to potentially be enhancing access to primary health care in those areas.^19^ An interprofessional assessment performed by an urban-based PT collaborating via videoconferencing with a rural Nurse Practitioner (NP) who can perform relevant portions of the ‘hands-on’ assessment with a patient, may be a solution to overcome the barriers of performing an effective remote examination and development of management strategies. Lovo Grona et al. (2017) examined this model of care in a single case study and determined it was feasible to provide a team based neuromusculoskeletal assessment for CBD using remote presence robotics in a remote Northern community.^20^ Lovo et al. (2019) examined the experience of participants with CBD who experienced a teams and technology model of care using telehealth. 93.7% were satisfied or very satisfied with the assessment, and qualitative analysis found improved access to clinical care for CBD and identified that technology and interprofessional practice were important considerations for the the team-based model of virtual care.^21^ What has not yet been determined, is whether a team of NP and PT joining by telehealth would make similar decisions regarding diagnosis and management as an in-person PT. This agreement is called concordance.^22^

### Objectives

The objectives of this study were to evaluate the concordance of diagnostic and management recommendations arising from: 1) a team consisting of a NP located with the patient and a PT joining via telehealth (NP/PT_team_), 2) an in-person assessment session with a PT only (PT_alone_), and 3) an in-person assessment with a NP only (NP_alone_).

## Methods

### Patient recruitment

Twenty-seven people with CBD were recruited from the Saskatoon area in the Canadian province of Saskatchewan. Inclusion criteria included: aged 18–80 years; low back and/or related leg symptoms which limited usual activities or daily routine, and the symptoms had to be present for at least 3 months. People were excluded if they had third-party payer insurance for their back-related complaints, if they had primarily neck or thoracic pain, or if there were language, reading, or comprehension barriers that would limit their ability to fully participate. All participants provided informed, written consent for their involvement. The study was approved by the Biomedical Ethics Board of the University of Saskatchewan, #12–340. This study was registered with ClinicalTrials.gov NCT02225535.^23^ A detailed study protocol has previously been published.^24^

### Health care professional groups

PT and NP are self-regulated professions in Canada. PTs are licensed in each province and have a Bachelor's or entry to practice Master's degrees. NPs have a Master's level degree and an advanced scope of practice, including ordering of diagnostic tests, prescription of medication and primary patient management.^25^ Although the NP model is not implemented in all countries and jurisdictions, in Canada a NP's scope of practice is advanced, as opposed to the scope of a nurse clinician.

Each participant underwent an assessment with all three groups: 1) in-person NP (NP_alone_); 2) a PT joining an in-person NP though telehealth (NP/PT_team_); 3) in-person PT (PT_alone_). The NP and PT on the team were different practitioners than the NP_alone_ and PT_alone_. Participants were instructed before the first assessment that they must not share any information learned from assessments with the subsequent practitioners, and subsequent practitioners reminded them of this requirement.

The NP and PT on the team were located in different rooms, and the patient was with the NP. The practitioners had 20 years of experience each. The team underwent interprofessional training on each other's competencies prior to the study, which consisted of review with the PT of neuromusculoskeletal assessment techniques and clinical reasoning specific to CBD, as well as NP considerations of subjective history, medication management and pain management history. The NP on the team performed hands-on components of the assessment such as deep tendon reflexes, muscle strength testing, dermatomal and nerve mobility testing. The NP_alone_ had > 30 years of experience. PT_alone_ had 10 years of experience. Both PTs in this study were trained and practiced at the same spinal triage service.^26^ The NPs did not practice at the same location.

### Measures

**Figure 1.**
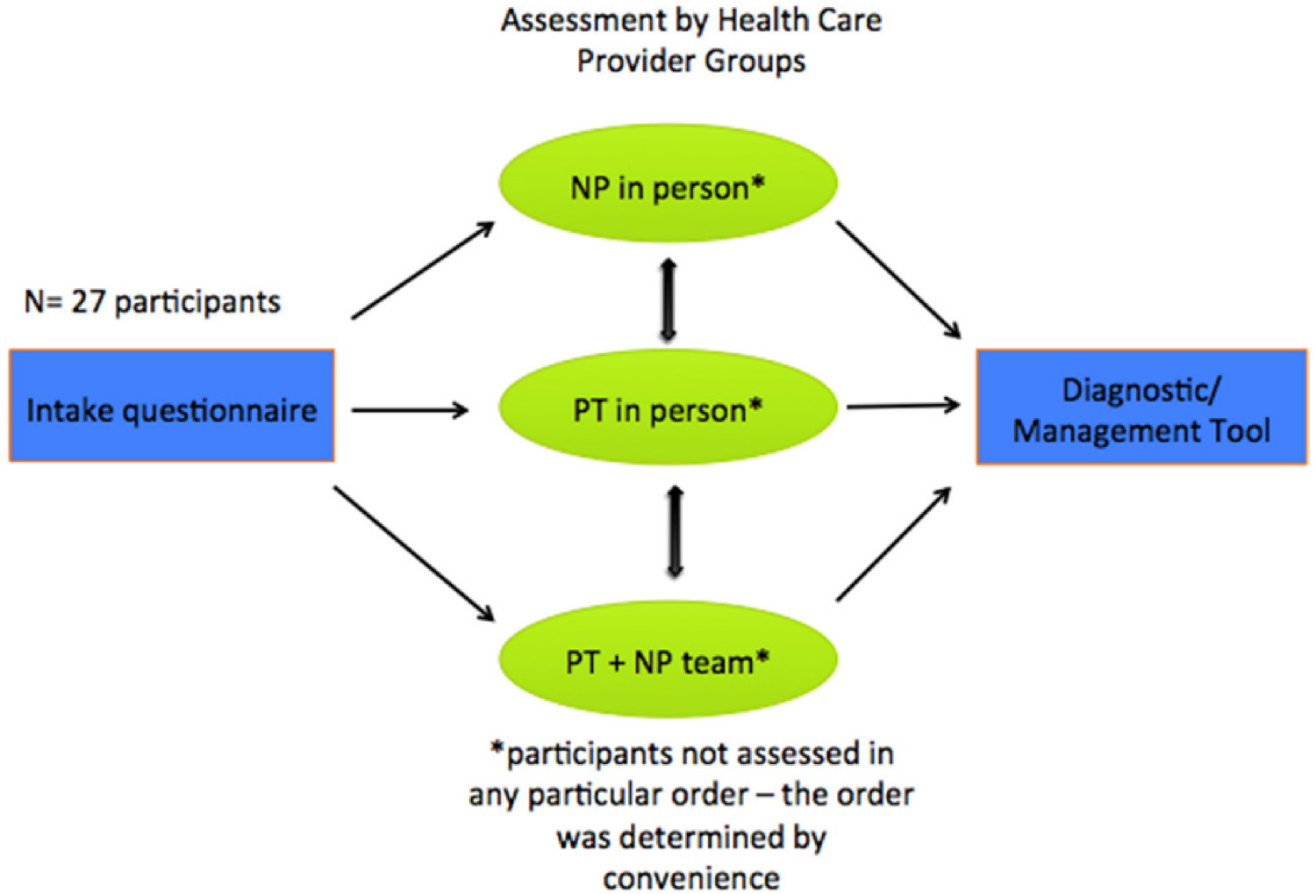
Overview of study design and timing of measures.

All participants completed an intake questionnaire that included: 1) personal and medical history; 2) length of time with pain; and 3) numeric pain rating scale.^27^ They also completed the Modified Oswestry Disability Questionnaire.^28,29^ Figure 1 demonstrates the flow of participants through the assessment groups and the outcome measures collected.

The time of assessment for each participant, for each assessment group was recorded. Each group (PT_alone_, NP_alone_ and PT/NP_team)_ completed an online password protected diagnostic classification tool that was previously developed and used in a spinal triage study,^30^ that was modified to capture an interprofessional management approach. The diagnostic classification tool was divided into two main components: diagnostic classification and management recommendations. Variables of diagnostic classification included: 1) Diagnostic Triage: “back pain”, “medical”, “mechanical other body part” or “spinal cord/cauda equina” as primary source of problem; 2) Low Back Pain Triage: “serious spine”, “nerve root”, “non-specific back pain/mechanical” or “not spine related”. Variables of treatment recommendations included: “no follow-up”, “urgent surgical”, “emergent surgical”, “PT/Rehab”, “surgical and PT/Rehab”, “other specialist”, “education and self-management”, “refer to primary for pharmaceutical management”, “imaging or other diagnostics”, “lab or other”. The categories represented nominal variables.

### Analysis

A descriptive analysis of the study sample and the assessment durations for each provider type/group was recorded. The Shapiro-Wilk Test was used to evaluate for normal distribution of variables. Parametric tests were used for normally distributed variables, and non-parametric tests for non-normally distributed variables. For those variables that were not normally distributed, median and interquartile range were presented, whereas normally distributed variables were described with mean and standard deviation. Duration of assessments was compared with independent t-test for PT_alone_ and NP_alone_ and with Wilcoxon's for PT_alone_ versus PT/NP_team_ and NP_alone_ versus PT/NP_team_.

Overall observed agreement for diagnostic and management categories was calculated as the proportion of cases on which the providers agreed. Level of agreement for diagnostic and management recommendation categories between each provider group (i.e. PT/NP_team_ vs. PT_alone_ and NP_alone_) was evaluated with kappa (k).^31^ Kappa compares category ratings (in this case diagnostic and management recommendation classifications) by independent evaluators and accounts for agreement due to chance. Category adjacency was considered and although some adjacency is inherent in clinical assessment, in this case categories were determined nominal rather than incremental, so weighted kappa was not used.^32^ The classifications of Bertilson et al. were utilized for the present study to interpret kappa values: “ <0 ‘no agreement better than chance’, 0–0.2 ‘poor’, 0.21–0.40 ‘slight’, 0.41–0.60 ‘moderate’, 0.61–0.80 ‘good’ and 0.81–1.0 ‘excellent’.^31^ Categories with zero or low cell counts were collapsed and re-coded as kappa is not appropriate to use in cases with zero cell counts or higher than 90% (or less than 10%) prevalence of an outcome. For diagnostic classification and treatment recommendations, three calculations were made on each group, thus, a Bonferroni correction was applied to set the alpha value at 0.017 (ie: 0.5/3).

It was pre-determined that a sample size of 22 patient participants was required to achieve 80% power at 0.60 kappa.^33^ The kappa sample calculation is based on two raters. A sample size calculation reference for more than two raters was not available.

## Results

### Participant demographics

Forty-nine potential participants were screened. Twenty-seven participants initiated and completed this study. Three cancelled due to resolution of symptoms, two cancelled due to illness, 14 had scheduling conflicts and three did not attend. Participant demographics are presented in Table 1. More than half of the participants were over age 50. Sixty-seven percent were overweight or obese as defined by Body Mass Index (BMI),^34^ and 70% were female. Fifty-six percent of participants worked full or part-time, while the remainder were students or retired. The median amount of time having low back pain was seven years. Moderate or severe disability (measured as values greater than 20 on the Oswestry Disability Index)^29^ was reported by 55.5% of participants.

**Table 1.** Participant demographic and health characteristics.

Characteristics	Frequency (%)	Descriptive Statistics
Age		Mean 53.7
<50 years	12 (44.4)	SD 18.1
≥50	17 (58.6)	Range 21–78
Sex – Female	19 (70.4)	
Male	8 (29.6)	
BMI (kg/m^2^)* categories^35^		Median 27.6
≥ 25.00 overweight	12 (44.4)	IQR 5.31
≥ 30.00 obese	6 (22.2)	
Work Status		
Full time	8 (29.6)	
Part time	7 (25.9)	
Students	2 (7.4)	
Retired	9 (33.3)	
Not answered	1	
Comorbidities		
Headache history	7 (25.9)	
Lung/breathing problems	3 (11.1)	
Heart problems	3 (11.1)	
Stomach problems	4 (14.8)	
Other bone/joint problem	20 (74)	
Time with Back Pain (years)		Median 7
< 7 years	11 (40.7)	SE 35.8
≥ 7 years	14 (51.9)	IQR 19.5
not reported	2	
NPRS		
Least		Mean 1.93
Most		Mean 7.70
Average		Mean 4.78
Modified Oswestry^29^		
Minimal (0–20%)	12 (44.4)	
Moderate (21–40%)	10 (37)	
Severe or greater (>41%)	5 (18.5)	

*BMI = Body Mass Index, SD = standard deviation, IQR = interquartile range, NPRS = Numeric Pain Rating Scale.

### Comparison between health care professional groups

NP_alone_ had 22 assessment durations recorded, PT_alone_ had 21, and NP/PT_team_ had 22 durations recorded. Recording errors account for the 5 times not recorded for NP_alone_ and NP/PT_team_, and 6 times not recorded for PT_alone_. The time spent by the NP_alone_ was normally distributed (mean 13.1 min, standard deviation 4.1), PT_alone_ was normally distributed (mean 36.0 min, standard deviation 8.5), and NP/PT_team_ was not normally distributed (median 31 min, interquartile range 13 min). Assessment durations for NP_alone_ and PT_alone_ were significantly different (*p *< 0.00) with NP_alone_ having a significantly shorter assessment duration. NP_alone_ had a significantly shorter duration than PT/NP_team_ (*p *< 0.00). PT_alone_ and PT/NP_team_ did not demonstrate a difference in time spent with participants (*p* = 0.24). Three high-duration outliers were noted for time spent with the PT/NP_team._ When these outliers were removed, the distribution for time spent with PT/NP_team_ was normally distributed (mean 29.4 min, standard deviation 6.40 min). When compared with NP_alone_ and PT_alone_ using a t-test, there was still a significant difference between PT/NP_team_ and NP_alone_ (t-test *p* < 0.00) and there was also a significant difference between PT/NP_team_ and PT_alone_ (t-test *p* < 0.00).

### Evaluation of concordance

Inter-rater percent agreements for diagnostic and management categories are described in Table 2. Overall, for diagnostic categorization, the lower back was determined to be the primary source of pain in 92.6% of cases by the PT_alone_, 85.2% by the NP_alone_ and 100% by the PT/NP_team_. Both PT_alone_ and NP_alone_ classified only 74% of the participants as presenting with mechanical back pain, whereas the PT/NP_team_ felt that mechanical back pain was responsible for 77.8% of cases. PT_alone_ and PT/NP_team_ felt that 7.4% of participants had a non-spine related concern, whereas NP_alone_ felt that 11.1% of participants had a non-spine related problem.

**Table 2.** Prevalence of diagnostic and management recommendations by group.

Variable	PT_alone_ n/27 (%)	NP_alone_ n/27 (%)	PT/NP_team_ n/27 (%)
Diagnosis*:			
Problem in back	25 (92.6)	23 (85.2)	27 (100)
Medical	0	0	2 (7.4)
Mechanical/Other body part	3 (11.1)	5 (18.5)	1 (3.7)
Spinal cord/ cauda equina	0	1 (3.7)	2 (7.4)
LBP Triage			
Serious spine pathology	0	1 (3.7)	2 (7.4)
Nerve root	5 (18.5)	3 (11.1)	2 (7.4)
Non-specific/mechanical	20 (74.1)	20 (74.1)	21 (77.8)
Not spine related	2 (7.4)	3 (11.1)	2 (7.4)
Treatment Recommendations:			
No further follow up	0	1 (3.7)	3 (11.1)
Urgent surgeon referral	1 (3.7)	1 (3.7)	2 (7.4)
Emergent surgeon referral	0	0	0
Referral to another specialist	2 (7.4)	1 (3.7)	3 (11.1)
PT Rehab	22 (81.5)	14 (51.9)	20 (74.1)
PT and Surgical Referral	2 (7.4)	2 (7.4)	0
Education and Self Manage	15 (55.6)	21 (77.8)	2 (7.4)
Refer Primary for Pharm	6 (22.2)	0	4 (14.8)
Imaging and diagnostic tests:	1 (3.7)	1 (3.7)	1 (3.7)
Lab or other	0	1 (3.7)	1 (3.7)

* determination of the source of the participant's symptoms, based on clinical examination.

The most common management recommendation made by all provider groups was “PT follow-up” (81.5% for PT_alone_, 51.9% NP_alone_, 71.4% for the PT/NP_team_). The PT_alone_ and NP_alone_ felt that 3.7% of cases (1 out of 27) required “urgent surgical referral”. The PT/NP_team_ referred 7.4% to “urgent surgical referral”. PT_alone_ referred 22.2% of participants back to their primary care provider for pharmacological workup, while the PT/NP_team_ referred back 14.8%, and the NP_alone_ did not refer any patients back for pharmacological workup. The authors note that the NP_alone_ has an autonomous practice, so it may not be surprising that the NP_alone_ did not need to refer patients back to a primary physician.

Level of agreement for diagnostic and management categories between each provider group is presented in Table 3. For the PT_alone_ and NP_alone_ comparisons, ‘good’ agreement (*k* = 0.63) was found for diagnosis of “back problem” and management recommendation of “urgent surgical referral” (*k* = 0.649). ‘Moderate’ strength in agreement was found for “overall categorization of diagnosis” (*k* = 0.474). ‘Slight’ strength in agreement was found for diagnosis of “medical/other body part mechanical or spinal cord lesions” (*k* = 0.348), and “referral back to primary practitioner for pharmacology/lab or imaging” recommendations (*k* = 0.372). All of these were found to be statistically significant agreements except for “medical/other body part mechanical or spinal cord lesions”.

**Table 3.** Inter-group diagnostic and management recommendation concordance.

Variable	PT_alone_ versus NP_alone_ Percent Agreement and Kappa (k)	NP_alone_ versus PT/NP_team_ Percent Agreement and Kappa (k)	PT_alone_ versus PT/NP_team_ Percent Agreement and Kappa (k)
CBD Triage Back Problem	**92.6%, k = 0.63 (p < 0.00)**	85.2%, k *	92.6%, k *
Medical/Mechanical Other/Spinal Cord Lesion	81.5%, k = 0.348 (*p* = 0.05)	81.5%, k = 0.43 (*p* = 0.024)	70.4%, k = −0.161 (*p* = 0.381)
Overall Categorization	**77.8%, k = 0.474 (*p* = 0.001)**	66.7%, k = 0.176 (*p* = 0.166)	**74.1%, k = 0.359 (*p* = 0.004)**
Management Recommendation No follow up	96.3%, k = *	85.2%, −0.059 (*p* = 0.719)	88.9%, k = *
Urgent surgical referral	**92.6%, k = 0.649 (*p* < 0.00)**	88.9%, k = 0.052 (*p* = 0.773)	**96.3%, k = 0.649 (*p* < 0.00)**
Referral to another specialist	88.9%, −0.052 (*p* = 0.773)	**92.6%, k = 0.471 (*p* = 0.004)**	81.5%, k = −0.098 (*p* = 0.603)
Referral to PT	59.3%, k = 0.063 (*p* = 0.683)	63%, k = 0.187 (*p* = 0.305)	**88.9%, k = 0.664 (*p* < 0.00)**
Refer to primary for pharmacology/lab/Imaging	**81.5%, k = 0.372 (*p* = 0.013)**	77.8%, k = 0.156 (*p* = 0.326)	66.7%, k = 0.090 (*p* = 0.639)

*unable to calculate kappa due to 0 count in cell (all 27 out of 27 participants were indicated to have back pain). Bold indicates agreement is significantly higher than expected by chance, with Bonferonni correction *p* = 0.017.

Comparing between NP_alone_ and PT/NP_team_, 85.2% agreement was found when identifying “the back” as the source of the problem. Kappa calculation was not possible in this case, as the PT/NP_team_ had zero participants identified without a back problem. Moderate agreement was found for a diagnosis of “medical/other mechanical body part or spinal cord lesion” (*k* = 0.43), and for “referral to other (non-surgical) specialists” (*k* = 0.471) but only “referral to other (non-surgical) specialists reached significance.

Comparison of PT_alone_ and PT/NP_team_ was 92.6% agreeable for back pain as the primary source of the participant's problem. Kappa cannot be calculated at this high level of agreement (i.e. > 90%).^31,32,36^ Strong agreement was noted for “urgent surgical referral” (*k* = 0.649), and “recommendation of any PT follow up” (*k* = 0.664). Slight agreement was found for “overall categorization of diagnosis” (*k* = 0.359).

## Discussion

The purpose of this study was to investigate the concordance (agreement), of diagnostic and management recommendations arising from three assessment groups: PT_alone_, NP_alone_ and PT/NP_team_ (PT joining with telehealth).

In the interprofessional assessment, NPs brought significant advantages to the team: rapport and trust with the patient, and knowledge of medical comorbidities as well as pain management approaches already tried. The consulting PT contributed consultative skills on neuromusculoskeletal assessment techniques, as well as clinical reasoning on the intricacies and management approaches for CBD.

The two PTs (PT_alone_ and PT_team_) were trained and practiced in the same setting with similar experience in spinal triage, and were accustomed to a similar assessment format. The PT/NP_team_ went through pre-assessment interprofessional training to standardize their assessment. The NP_alone_ followed their traditional assessment approach. The NP_alone_ and NP working with the PT in a team had different practice settings and years of experience. The differences in clinical practice may have contributed to overall findings, however the most likely reason for differences is the presence of the PT on the interprofessional team.

The present study noted strong agreement between PT_alone_ and NP_alone_, as well as PT_alone_ and PT/NP_team_ with respect to “overall diagnoses” and management decisions such as “need for urgent surgical referral” and “follow up PT”. Although concern has been expressed that elements of a conventional face-to-face physical examination require the PT to be “hands-on” with the patient,^18^ in the present study adapting a neuromusculoskeletal assessment to an interprofessional approach of the NP/PT_team_ resulted in similar outcomes for “overall diagnosis” and “management recommendations” as an in-person PT assessment.

PT_alone_ and NP_alone_ comparisons had ‘good’ agreement on the management recommendation for “urgent surgical referral”, and ‘moderate’ strength in agreement for “overall categorization of diagnosis”. NP_alone_ and NP/PT_team_ had 85.2% agreement for identification of “the back” as the primary source of the participant's problem, but had lower agreement on overall diagnosis or recommendation for “urgent surgical management”. PT_alone_ and PT/NP_team_ were 92.6% in agreement that the back was the source of participants’ problems, and ‘strong’ agreement was noted for “urgent surgical referral”, and “recommendation of any PT follow up”.

Participants spent a mean of 13.1 min with the NP_alone_, 36 min with the PT_alone_, and 31 min with the PT/NP_team_. NP_alone_ assessment durations were significantly less than both PT_alone_ and NP/PT_team._ This may be an important factor in the consideration of a new model of care. The increased length of assessment durations noted with the NP/PT_team_ compared to NP_alone_ would come with increased cost for the healthcare system. However, this refers only to the cost of clinician time, without reference to travel or time patient cost, which are important future considerations since they may represent cost savings to the patient. The present study did not collect information on direct cost savings to the patient which may have been realized due to the team model, for example time and travel already noted, as well as the potential cost savings due to identification of referral needs in the case of medically complex patients, which occurred in the team model and is discussed in detail below. The systematic review by Wade et al. found that medical and specialist care models using telehealth for rural outpatient services also identified studies where telehealth was determined more expensive than the control (in terms of costs of health care services).^37^ To the best of our knowledge, there are no cost analyses for PT involvement in an interprofessional care model using videoconferencing.

In terms of longer assessment times on the NP/PT_team_, there were three outliers out of 22 (71, 65 and 55 min), which may provide valuable cases to demonstrate the enhanced effectiveness of a team approach. In these cases, the NP/PT_team_ identified complex medical concerns. In the first case, they referred to geriatric assessment unit for medical review, occupational therapy and PT for reconditioning and treatment for mechanical degenerative pain (of the lumbar spine). PT_alone_ recommended that individual treatment by a lone practitioner would not likely be effective, and that the patient required a team approach to care. NP_alone_ recommended return to physician for workup, and noted the importance of social connections and psychological treatments to be ongoing. In the second outlier, NP/PT_team_ recommended full cardiac, neurological and metabolic workups with the primary provider due to reported: excessive thirst, diminished bilateral foot sensation, noted dyspnea/wheeze, deconditioning, diaphoresis, history of syncope, and diminished memory. NP_alone_ recommended “referral back to primary provider” due to concerns of generalized weakness, proprioceptive and sensory difficulties. PT_alone_ recommended possible candidate for facet injection. In the third outlier case, the NP/PT_team_ reported widespread irritable capsular restrictions including bilateral hips and cervical spine and recommended PT follow-up. NP_alone_ recommended self-care with videos on posture, as well as continued exercises given by the patient's own PT. PT_alone_ recommended PT follow-up and self-care for the lumbar pain. However, the third case was more likely indicative of osteoarthritic flare up and involved screening of hips and cervical spine to confirm; this additional screening required a greater length of time.

The present study examined a full, comprehensive neuromusculoskeletal assessment process and clinical reasoning process for the management of CBD. Previous research using telehealth for back pain has focused on specific assessment techniques, or portions of assessments.^38,39^ Other studies have examined a single case of neuromusculoskeletal assessment^20^ and have focused on experience of patients and practitioners with this type of care.^21^ No previous studies have examined the concordance, or level of agreement, of a full neuromuscular assessment for CBD and associated clinical decision-making in comparison to a reference standard of in-person PT assessment.

Reduced access to PT in rural and remote regions is a known healthcare disparity. Bath and Janzen found that patients appreciated the expertise and education provided by an in-person PT for their back pain, but that this did not solve the problem of limited rural access to PT.^15^ Briggs et al. interviewed rural Australian people with CBD. They found that some of the participants expressed dissatisfaction with limited access to both interdisciplinary care and practitioners with experience managing pain. Participants raised the idea of telehealth technologies for improving care.^40^ The present study demonstrates the feasibility of bringing a PT consultant to a rural team for CBD assessments using telehealth, which could improve access to PT in rural areas. The NP/PT_team_ decisions about overall diagnosis, referral to surgeon and referral for PT showed adequate agreement with the decisions of a PT_alone_, which means similar decisions were made through the team assessment over telehealth, as would have been made if a PT_alone_ assessed the participants.

### Limitations

There were several limitations to this study. Participants underwent assessment by all three professional groups in one day. This may have contributed to patient fatigue or exacerbation of pain, especially in the more compromised patients with comorbidities. Also, order of assessment was determined by convenience due to lack of space and availability of professionals, so randomization and sequencing to the three assessment scenarios was not feasible. It is possible that although participants were instructed not to do so, they may have behaved differently or been sensitized to questioning in subsequent sessions due to learning of assessment approaches and questions. This potential effect was not counterbalanced by random presentation of conditions.

This model is based on an interprofessional team consisting of a PT and a NP. In Canada, the NP is a self-regulated professional with advanced scope of practice. NPs are able to order diagnostics, prescribe medications and perform other skills that are not within the scope of a nurse clinician. This may limit the generalizability of these findings to teams with similar health practitioner scopes of practice. For example, in other jurisdictions around the world there is not a NP role, or regulatory allowances for this role differ. In the United States for example, there is not consistency among all of the states as to what advanced roles an NP is allowed to perform with the scope of their duties.^41^ The findings would not necessarily be transferable to jurisdictions without advanced scope roles such as a NP.

The diagnostic and management concordance tool for CBD is a previously used instrument with operational definitions.^42^ However, it is a complex tool with many possible categories and it is possible that the practitioners could vary in their interpretations despite the operational definitions and training prior to its use. For example, “education and self-care” was selected a number of times by PT_alone_ and NP_alone_, but rarely by the PT/NP_team_. This may indicate that the NP/PT_team_ included this information as part of the assessment and discussion with the participant, rather than considering it as a separate recommendation.

Cohen's kappa is based on comparisons of two raters.^32^ In this study we had three groups to compare, so repeated calculations were done between pairs. Kappa cannot be utilized when one of the options had a “0” cell count, which happened when the PT/NP_team_ selected all 27 participants as having back pain as their primary problem. Both the NP_alone_ and PT/NP_team_ agreement for “back pain” diagnosis were very high (over 80% and 90% respectively) but (k) could not be calculated.

## Conclusion

This study evaluates the concordance or agreement of a team and virtual care approach for CBD with more traditional forms of single-provider in-person care for CBD. The ‘team’ consisted of a PT joining a NP and patient using telehealth. There was 92.6% agreement for PT/NP_team_ and PT_alone_ with respect to a back pain diagnosis, as well as ‘strong’ agreement for recommendations of “urgent surgical follow-up” and “referral to PT” management needs following lumbar neuromusculoskeletal assessment. The team model using telehealth was an effective model of care in this study. It is an option for integrating a PT into a care team, in comparison to PT_alone_, that may be suitable for rural and remote primary care teams who manage CBD. Decisions of diagnosis and management are in strong agreement with those made by PT_alone_. The addition of a team to provide care in comparison to a PT_alone_ or NP_alone_ appeared to result in unique management considerations for three complex cases with co-morbidities. This may suggest enhanced quality of care for patients with multiple co-morbidities. Future research should focus on evaluating the feasibility and effectiveness of this new model of care in a rural/ remote setting, ideally through randomized controlled trials, to compare different models of care on health and systems outcomes for patients with CBD. Future research should also include other primary practitioners working with the PT and patient – for example nurse clinicians and physicians.
